# Integrative Analysis of Genomic and Clinical Data Reveals Intrinsic Characteristics of Bladder Urothelial Carcinoma Progression

**DOI:** 10.3390/genes10060464

**Published:** 2019-06-17

**Authors:** Bin Zhou, Rui Guo

**Affiliations:** 1 School of Life Science, Tsinghua University, Beijing 100084, China; 2 Department of Biochemistry and Molecular biology, Shanxi Medical University, Taiyuan 030001, China

**Keywords:** bladder cancer, survival analysis, gene co-expression, genomic variation, microRNA-gene regulatory network

## Abstract

The progression of bladder cancer is generally a complex and dynamic process, involving a variety of biological factors. Here, we aimed to identify a set of survival-related genes that play an important role in the progression of bladder cancer and uncover their synergistic patterns. Based on the large-scale genomic profiling data and clinical information of 404 bladder cancer patients derived from The Cancer Genome Atlas (TCGA) database, we first discovered 1078 survival-related genes related to their survival states using univariate and multivariate Cox proportional hazardous regression. We then investigated the dynamic changes of the cooperative behaviors of these 1078 genes by analyzing their respective genomic features, including copy number variations, DNA methylations, somatic mutations, and microRNA regulatory networks. Our analyses showed that during the progression of bladder cancer, the biological disorder involving the identified survival-related genes can be reflected by multiple levels of abnormal gene regulation, ranging from genomic alteration to post-transcriptional dysregulation. In particular, the stage-specific co-expression networks of these genes undergo a series of structural variations. Our findings provide useful hints on understanding the underlying complex molecular mechanisms related to the evolution of bladder cancer and offer a new perspective on clinical diagnosis and treatment of bladder cancer.

## 1. Introduction

Bladder cancer is one of the most common tumors in human urinary systems, and its incidence lies in the forefront of the global cancer spectrum. Although the etiology of bladder cancer has been shown to be multifactorial and complicated, smoking and occupational exposure to chemical carcinogens are considered the main cancer-inducing factors [1]. In addition, familial inheritance can be another important causal factor [2]. According to the clinical pathological classification, bladder urothelial carcinoma (BLCA) accounts for the majority of bladder cancer.

The elucidation of the underlying molecular mechanisms of tumor evolution is one of the most important questions in cancer biology. The recent advent of high-throughput DNA sequencing has enabled researchers to profile the genomic features in the dysregulation of gene expression under various biological/clinical conditions. For example, it has been revealed that copy number variations (CNVs) can act as an important driving force in several cancers; such genomic variations can alter gene expression, and thus, affect the corresponding biological functions [3]. DNA methylation, an essential epigenetic mechanism, can regulate gene expression by modifying the CpG sites in DNA promoter regions. In bladder cancer cells, abnormal DNA methylation levels have been shown to be associated with the disorder of certain gene functions, and thus, contribute to the progression of bladder cancer. For instance, the hypermethylation in the promoters of *ITIH5* and *RBBP8* can facilitate the progression of bladder cancer [4,5]. In addition, somatic mutations are often considered as another important driving force in the progression of bladder cancer, especially for those genes involved in important intracellular signaling pathways. For example, Nickerson et al. [6] identified the mutational landscape related to the drug response in bladder cancer cell lines. In addition to the above variations at DNA levels, microRNAs can also play an important role in regulating the expression of mRNAs. Recent studies have revealed that microRNAs can be highly connected to the evolution of bladder cancer [7,8].

In this study, we aimed to reveal the underlying genomic characteristics of bladder urothelial carcinoma progression and understand how they may affect the survival of cancer patients. We first used a specific survival analysis method to discover 1078 survival-related genes that are highly related to the survival of 404 bladder cancer patients derived from the TCGA cohort. We found that, during the progression of bladder cancer, the stage-specific co-expression networks of these genes undergo a series of structural variations. To further reveal the potential intrinsic causes behind this phenomenon, we also investigated related genomic profiles from multiple perspectives, including copy number variations, DNA methylations, somatic mutations, and microRNA regulatory networks. In addition, we identified the important functional gene modules that are the most correlated with tumor staging and observed that they display significantly distinct association patterns with other independently measured genomic profiles, such as copy number variations and somatic mutations. These results can help to understand the underlying molecular mechanisms responsible for the evolution of bladder cancer and reveal potential biomarkers for clinical prognostic evaluation or therapeutic purposes of bladder cancer.

## 2. Materials and Methods

### 2.1. Raw Datasets

The genomic profiles and clinical information of BLCA patients were mainly downloaded from “National Cancer Institute GDC Data Portal Legacy Archive”. In particular, the RNA-seq datasets of BLCA patients contained 404 samples and all the gene expression values were normalized. The TCGA level 2 data of somatic mutation data in mutation annotation format (MAF) files were used. The TCGA level 3 methylation data were downloaded from “jhu-usc_BLCA.HumanMethylation450”. The TCGA level 4 correlations between mRNA expression and DNA methylation were obtained from the Broad GDAC Firehose. The TCGA level 4 copy number variation (CNV) data were downloaded from the Broad GDAC Firehose, including the gene-level table of copy number values and its discrete indicators as following: serious deletion = −2; deletion = −1; no change = 0; amplification = 1; high amplification = 2. We chose to use the “reads per million miRNA mapped (RPM)” from the TCGA level 3 microRNA quantification files as the microRNA expression values. 

The available public dataset GSE13507 (expression profiling of 165 primary bladder cancer samples by microarrays) was downloaded from the Gene Expression Omnibus (GEO) database for external validation. In this dataset, for those genes with multiple probes, we chose the average expression value. Besides that, according to the clinical information, we collected the tumor grade and invasiveness of each patient, and we found that the invasiveness was positively associated with tumor grade in 129 patients, whereas 36 patients had the opposite effect.

### 2.2. Cox Proportional Hazards Regression and Kaplan–Meier Analysis

We applied both univariate and multivariate Cox proportional hazards regression models to identify survival-related genes that may affect the survival of BLCA patients. The expression values of individual genes in all BLCA samples were first normalized according to their z-scores. We removed those genes with expression in <20 samples.

In univariate Cox proportional hazards regression, gene expression values were used as the only predictor variables, while in multivariate Cox proportional hazards regression, age, gender, tumor stage, and gene expression values were used as the predictor variables. The *p-*values were adjusted using the “Benjamini and Hochberg” method. With respect to the threshold of statistical significance for survival analysis, we used *p*-value < 0.05 and false discovery rate (FDR) < 0.1 for univariate Cox regression, and *p*-value < 0.05 and FDR < 0.05 for multivariate Cox regression. For all Cox regression models, we also checked the proportional hazards (PH) assumption. From the results of the PH test, the significant *p*-value (<0.05) indicated that the proportional hazards assumption was not satisfied for the corresponding variable. We excluded the 99 genes that violated this condition.

For the Kaplan–Meier survival analyses, we first divided all the BLCA samples into high and low groups according to the median DNA methylation beta values of individual selected genes. After that, we plotted the Kaplan–Meier survival curves and ran the log–rank test to compare the difference between two groups. Here, survival analysis was performed using the R package “survival”.

### 2.3. Gene Ontology (GO) Enrichment Analyses

The functional annotations of the selected genes and their gene ontology (GO) enrichment analyses were performed in DAVID v6.8 [9]. The threshold *p-*value < 0.05 was used to select the GO terms.

### 2.4. Construction of the Gene Co-Expression Networks and Detection of Functional Gene Modules

Based on the correlations of the gene expression values of the identified 1078 survival-related genes, we constructed their gene co-expression networks using the weighted correlation network analysis (WGCNA) algorithm [10]. Compared to the hard threshold filters, the WGCNA algorithm is a robust technique that can keep all the information of target genes and their relationships through a soft threshold method. To obtain the signs of the correlations between genes, we chose the “signed” type of the adjacency matrix based on the correlations of the 1078 genes. With the function “pickSoftThreshold” in the program, we picked the appropriate soft threshold *β* = 8 to construct the genes co-expression networks of these 1078 genes for all BLCA samples.

The gene modules were identified through the “cutreeDynamic” function. The minimum module size was set to 20. The heatmap of module–trait associations was generated using the “labeledHeatmap” function.

### 2.5. Preprocessing of Copy Number Variation Data

We obtained the CNV data from “SNP6 copy number analysis (GISTIC 2)” at the Broad GDAC Firehose (level 4) to perform our analysis. Here, we obtained the CNV data of 1018 survival-related genes (belong to 1078 survival-related genes) for 400 BLCA samples, including 129 samples for stage I/II, 139 samples for stage III, and 132 samples for stage IV (Table S6). For each gene, we counted the frequency of samples with CNVs (i.e., amplification or deletion) in every stage. Considering the imbalance in the number of samples in different tumor stages, we also used stage I/II as a baseline to normalize the frequencies in individual stages.

### 2.6. Preprocessing of DNA Methylation Data

From the Broad GDAC Firehose-“Correlation between mRNA expression and DNA methylation”, we obtained 933 DNA methylation probes for the identified 1078 survival-related genes and each of them were most anti-correlated with the expression value of the corresponding gene. We then extracted the *β*-values of these DNA methylation probes from the “jhu-usc.edu_BLCA.Human-Methylation450” files at TCGA. After that, we calculated the standard deviation (SD) of each DNA methylation probe, and then set 0.15 as the threshold of SD to select 340 active methylated genes. The reasons for such a setting were as follows: (1) the methylation variation of each probe should not be too low(greater than 0.1); (2) we calculated the quartiles of the 933 probes’ SD: 0.0473 (25%), 0.116 (50%) and 0.176 (75%). The threshold (>0.15) can cover the top 25%~50% probes with largest variations, which is a suitable scale.

We then applied the regularized Cox regression, a LASSO-based regression method, to identify a set of optimal genes with low multi-collinearity from the above 340 DNA methylation probes. In detail, we ran 10-fold cross validation to determine the optimal value of the regularization parameter, and 26 DNA methylation probes with non-zero coefficients were chosen through this analysis approach. For the risk score, we multiplied the coefficient of each gene with the corresponding beta value, and then summed them up as the score of each patient. This analysis was performed mainly through the “glmnet” R package.

### 2.7. Preprocessing of Somatic Mutation Data

After downloading the somatic mutation data from TCGA (level 2), in total, we obtained 6052 somatic mutations for 908 of the key 1078 genes in 397 BLCA samples, including 129 samples for stage I/II, 135 samples for stage III, and 133 samples for stage IV. We removed the silent mutations and retained other types of mutations, including missense mutations, nonsense mutations, frame shift deletion/insert, translation start site mutations, splice site mutations, nonstop mutations, in frame insert/deletion, etc.

### 2.8. Preprocessing of MicroRNA–mRNA Interaction Data

We first obtained all the experimentally validated interactions between microRNAs and the selected 1078 survival-related genes from the miRWalk2.0 database [11]. Next, for each stage of bladder cancer, we calculated the correlation coefficients between the expression values of the 1078 genes and the corresponding interacting microRNAs. If the correlation coefficient between a pair of microRNA and gene was less than −0.3, we regarded them as a possible regulatory pair, otherwise the corresponding link was removed from the original microRNA–gene interaction network. In addition, we retrieved those specific microRNAs that had been reported to be associated with bladder cancer from the miRCancer database (version of December 2016) [12].

### 2.9. Network Analyses of MicroRNA Regulatory Networks

We used the R package "igraph" to calculate the assortative degrees of microRNA regulatory networks in different stages of bladder cancer. Assortativity degree is a concept in network science which measures the level of homophily of a graph, according to a specific vertex labeling schema (i.e., assigning some values to vertices). If the assortativity degree is high, it means that the connected vertices tend to have the same labels or similar assigned values. In other words, a pair of connected nodes with similar degrees tend to have an assortativity (i.e., positive) relationship. On the other hand, a pair of connected nodes with largely different degrees tends to have a disassortativity (i.e., negative) relationship. The network graphs were generated by Cytoscape 3.5.0.

### 2.10. Ordinal Logistic Regression for Integrative Analysis

We used the “mnrfit” function in Matlab 2016b to perform the ordinal logistic regression task. In this integrative analysis, the response variable was the tumor stage (stage I/II = 3, stage III = 2, stage IV = 1), while the predictor variables included the average expression values (z-score normalized) of protective and hazardous genes, the frequency of copy number amplifications and deletions (z-score normalized), the risk score of DNA methylation, age, and gender (Male = 0, Female = 1).

## 3. Results

### 3.1. Overview of Our Analysis Pipeline

**Figure 1:**
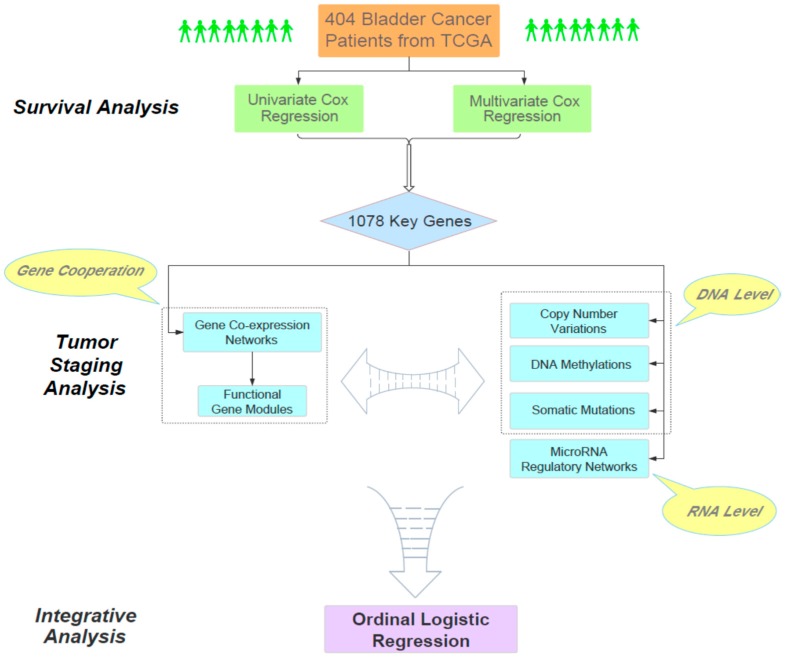
The flowchart of our analysis pipeline.

The main goal of our work aimed to find the molecular clues for understanding the relationship between the overall survival time of bladder cancer patients and their pathological staging. Overall, our analysis consisted of three phases (Figure 1). First, through large-scale Cox regression models (i.e., univariate and multivariate Cox regression), we identified 1078 survival-related genes according to their impact on the survival status of 404 bladder cancer patients obtained from TCGA. As a bridge connecting survival and tumor staging, the cooperative patterns of these survival-related genes in different stages were particularly analyzed. Next, we focused on these 1078 genes and analyzed the relationships between the progression of bladder cancer and several related genomic factors, including gene co-expression networks, functional gene modules, copy number variations, DNA methylations, somatic mutations and microRNA regulatory networks. Finally, we performed an integrative analysis on the joint effect of multiple biological factors on the staging of bladder cancer. Overall, our study provided a systematic and reasonable pipeline to integratively analyze the genomic and clinical data and reveal the intrinsic genomic characteristics of the progression of bladder cancer.

### 3.2. Identification of Survival-Related Genes Based on Cox Regression Modeling

As a useful statistical method, survival analysis can explore the links between survival states and different potential influential factors. Here, we applied both univariate and multivariate Cox proportional hazards regression models to select a set of survival-related genes that may have an important effect on the survival of BLCA patients (see Materials and Methods section). In particular, in univariate Cox regression, we used the measured expression values of individual genes as the only predictor variables. Initially, we obtained the expression values of 19,472 genes for all 404 BLCA patients, after removing genes that were rarely expressed (i.e., <20 samples). We then chose 1307 candidate genes based on the thresholds’ *p*-value < 0.05 and the false discovery rate (FDR) < 0.1. Next, we checked whether the proportional hazards assumption held for individual candidate genes and excluded 99 genes that did not satisfy this assumption (see Material and Methods section). That is, overall, the univariate Cox regression analysis yielded 1208 candidate genes. In multivariate Cox regression, in addition to the expression values of the above resulting 1208 genes, we also integrated clinical information (Table S1), including age, gender, and tumor stage information (stage I/II = 3, stage III = 2, stage IV = 1) of BLCA patients as input predictor variables. We used the FDR threshold < 0.05 and also checked the proportional hazards assumption to further screen the candidate genes. In the end, we obtained 1078 candidate genes (Table S2) from multivariate Cox regression, which were defined as survival-related genes and then used for the downstream analysis. 

**Figure 2:**
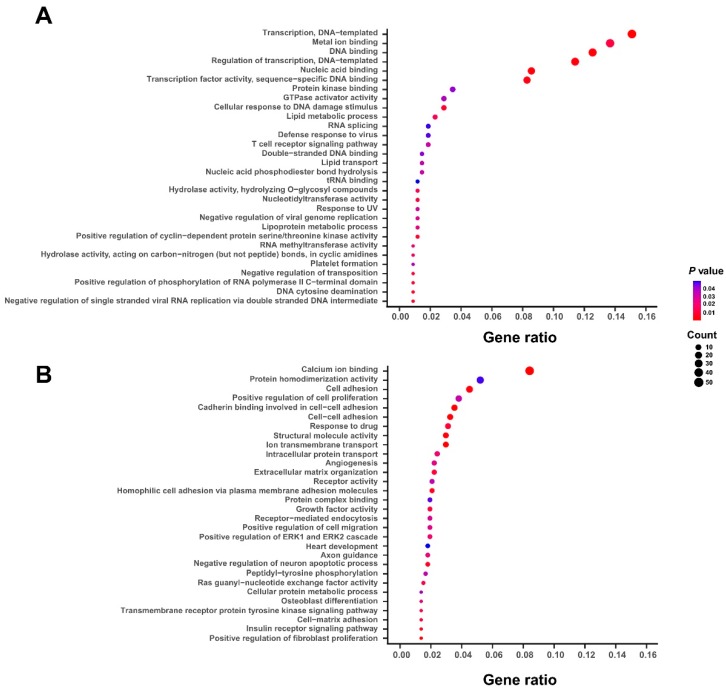
The functional enrichment analyses of 1078 survival-related genes. The gene ontology (GO) enrichment analyses of protective (**A**) and hazardous (**B**) genes in the selected set of 1078 survival-related genes that are important for the survival of BLCA patients. The GO terms were ranked according to the ratios of the involved genes and the top 30 significant GO terms with *p*-values < 0.05 are shown here.

According to the coefficients of gene expression derived from the above multivariate Cox regression model, the 1078 survival-related genes could be divided into two groups: 356 genes with negative coefficients and 722 genes with positive coefficients, which were defined as protective and hazardous genes, respectively (Table S2). To characterize the potential biological functions of the above selected survival-related genes, we performed the gene ontology (GO) enrichment analysis on both protective and hazardous genes. We found that the GO terms of protective genes are mainly enriched in the fundamental cellular processes or functions, such as nucleic acid binding, RNA splicing, and tRNA binding (Figure 2A). On the other hand, the hazardous genes are more possibly involved in the pathogenesis of bladder cancer, such as cell adhesion, angiogenesis, response to drug, and positive regulation of cell migration (Figure 2B). Overall, these functional enrichment analyses indicated that the selected 1078 survival-related genes, especially for those hazardous ones, were tightly associated with the biological functions related to the progression of bladder cancer.

### 3.3. The Heterogeneity of Gene Co-Expression Patterns in Bladder Cancer Patients at Different Tumor Stages

**Figure 3:**
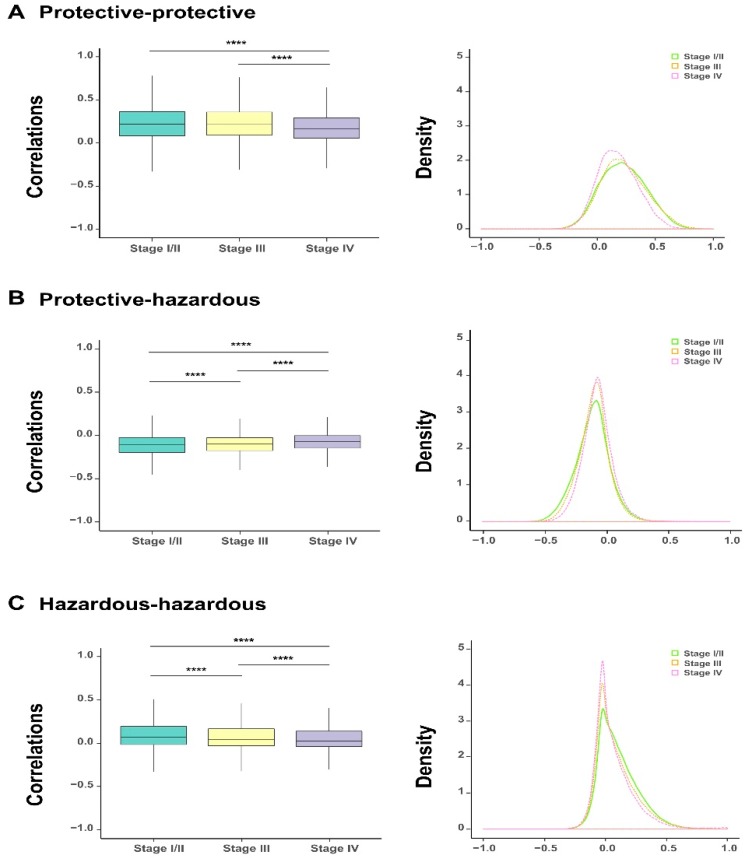
The dynamic changes of correlations between the selected 1078 survival-related genes for bladder urothelial carcinoma (BLCA) patients at different cancer stages. The boxplots on the comparisons of correlation coefficients (all outliers are not shown) and the corresponding density curves between protective and protective genes, protective and hazardous genes, and hazardous and hazardous genes, respectively (**A**–**C**). * *p*-value < 0.05; ** *p-*value < 0.01; *** *p*-value < 0.001; **** *p-* value < 0.0001; two-sided Wilcoxon rank–sum test.

The above survival analysis has divided the 1078 survival-related genes into two groups, including protective and hazardous genes. To investigate the correlation between the expression of genes within or between these two gene groups during the evolution of bladder cancer, we analyzed the correlation coefficients of expression values between protective and protective genes, protective and hazardous genes, and hazardous and hazardous genes at each tumor stage. The comparisons showed that as the severity of bladder cancer enhanced (i.e., along the order of stage I/II, stage III, and stage IV), a significant decline was observed in the correlations within either homogeneous genes (i.e., between protective and protective or hazardous and hazardous genes) or heterogeneous ones (i.e., between protective and hazardous genes) (Figure 3A–C). This change can also be reflected in the shifts of the corresponding density curves, i.e., the density curves became higher and narrow along with the progression of bladder cancer. Taken together, the above analysis results indicated that the alteration in the expression of the identified survival-related genes were closely related to the progression of bladder cancer, which also evidently reflected the heterogeneity of their co-expression patterns.

### 3.4. Construction of Co-Expression Networks of Survival-Related Genes and Functional Gene Modules Detection

Gene co-expression networks can provide an overall scenery of gene–gene associations. Based on the gene expression values of BLCA patients at different stages, we constructed the stage-specific gene co-expression networks using the WGCNA algorithm [10], which has been widely used in studies of weighted correlation networks (see the Materials and Methods section).

**Figure 4:**
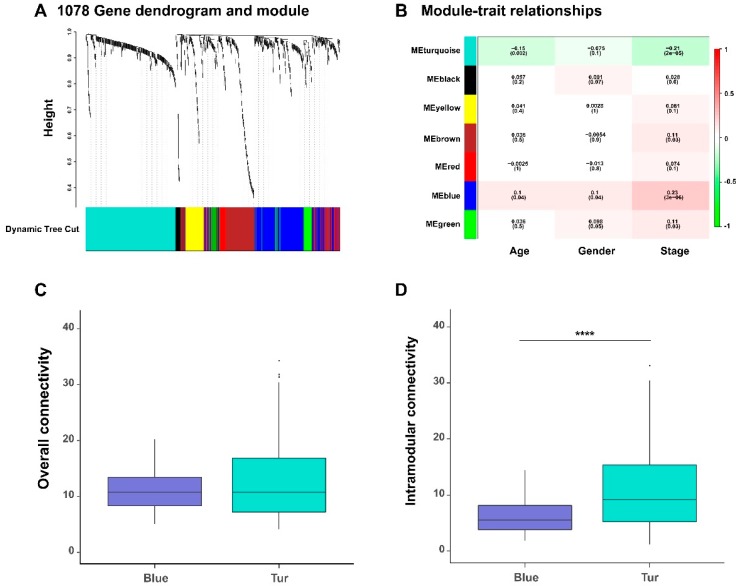
The functional modules of the gene co-expression networks detected by the weighted correlation network analysis (WGCNA) algorithm. (**A**) The hierarchical clustering tree (i.e., dendrogram) based on the dissimilarity scores and the individual gene clusters resulting from the dynamic tree cut algorithm. (**B**) The relationships between the module eigengenes (rows), which are defined as the first principal components of the gene expression profiles within individual modules, and the clinical traits (columns) of all BLCA patients. Each box shows the correlation coefficient and the corresponding *p*-value (in the bracket). The colors on the left represent individual functional gene modules, corresponding to those identified in (**A**). The comparison of the overall connectivity (**C**) and the intramodular connectivity (**D**) of members in blue and turquoise modules. **** *p-*value < 0.0001; two-sided Wilcoxon rank–sum test.

In a gene co-expression network, its network modules can provide useful hints for understanding the biological functions of the involved genes. We detected seven functional gene modules from the previously constructed gene co-expression networks by dynamic tree cut algorithm from the WGCNA method (Table S3; also labeled in different colors in Figure 4A).

To identify gene modules related to the clinical traits of BLCA patients, we also calculated the correlation coefficients between the module eigengenes (which are defined as the first principal components of gene expression profiles of the corresponding modules) and the clinical traits of cancer patients (Figure 4B). As tumor progression is generally closely related to the survival of patients, we particularly looked into those gene modules that were associated with the tumor stages. We observed two gene modules (labeled in blue and turquoise colors in Figure 4, respectively), were significantly negatively and positively correlated with the stages of bladder cancer. In addition, we also found that the majority (93%) of genes in the turquoise module (i.e., negatively correlated with the stages) were protective, while all genes in the blue module (i.e., positively correlated with the stage feature) were hazardous.

We further calculated the overall connectivity (the average degrees of nodes over the whole network) and the intramodular connectivity (the average degrees of nodes within the module) of the members in blue and turquoise modules (Table S4). We found that, the members of two network modules have different clustering behavior characteristics in local and overall levels; the genes in the turquoise module had stronger correlations with each other than those in the blue modules, which also reflected the difference of co-expression patterns between the two network modules (Figure 4C–D).

We next looked into those genes with the top 30 intramodular connectivity degrees for each network module and found that some of them had been reported in the literature to be associated with bladder cancer. For instance, *PDGFRB* was proven to be closely related to the recurrence of non-muscle-invasive bladder cancer [13]. *MARVELD1* was found to be downregulated in several cancer types including bladder cancer [14]. *KCNE4,* an ion channel gene, was found to show abnormal expression level in bladder cancer samples [15]. It was shown that the expression of *CPT1B* was downregulated in bladder cancer tissues together with other genes in the carnitine–acylcarnitine metabolic pathway [16]. In addition, *CDK6* was proven to be involved in the regulatory pathways in bladder cancer [17]. 

Overall, these observations implied that the genes with high-connectivity degrees in the network modules may also have important biological functions in the progression of bladder cancer. Taken together, the above results indicated that the associations between the survival of BLCA patients and their tumor stages can be reflected by the different group behaviors of the survival-related genes.

### 3.5. The Effect of Copy Number Variations on Genomic Stability of Survival-Related Genes in the Progression of BLCA

**Figure 5:**
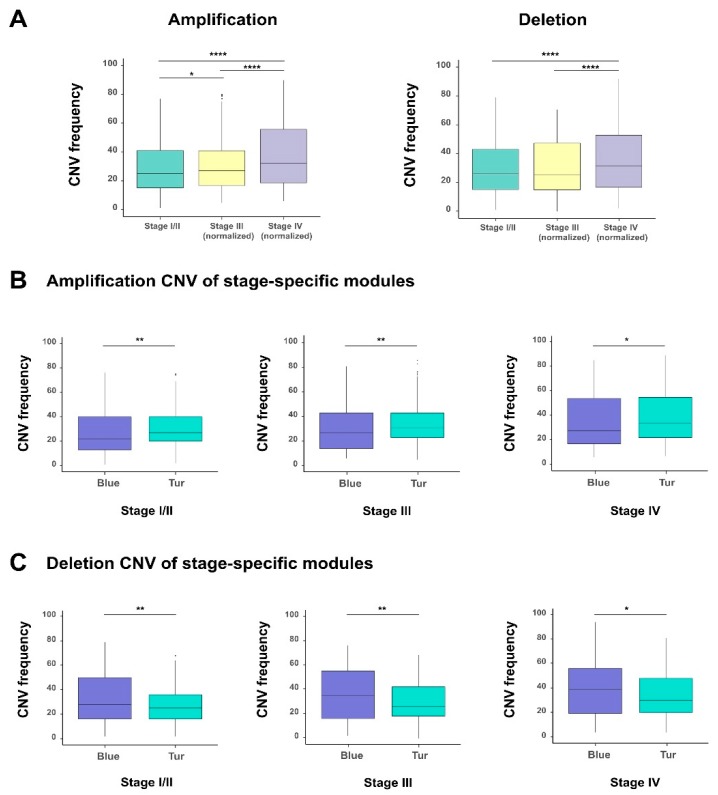
The analysis of copy number variations (CNVs) in different stages of bladder cancer. (**A**) The comparison of CNVs, including amplification and deletion between different stages of bladder cancer. The comparison of amplification (**B**) and deletion (**C**) of CNVs between blue and turquoise modules (corresponding to those two groups identified in Figure 4, which were most positively and negatively correlated with the tumor stage feature, respectively) in all and stage-specific samples. * *p*-value < 0.05; ** *p*-value < 0.01; *** *p*-value < 0.001; two-sided Wilcoxon rank–sum test.

Copy number variations (CNVs) refers to the deletion or amplification of DNA fragments in chromosomes and can greatly influence the expression of related genes. Here, we analyzed the difference of CNVs in individual tumor stages for the 1018 survival-related genes selected from the previous survival analysis (see Material and Methods; Table S5). The comparison showed that individual stages of bladder cancer displayed significantly different CNV frequencies, and both amplification and deletion increased remarkably along with the progression of bladder cancer (Figure 5A). Besides that, to explore the effect of copy number variations on the expression of these 1018 survival-related genes, we calculated the Pearson correlation coefficient between each gene expression and its corresponding copy number value for all samples. We found that 341 genes showed the correlation coefficients > 0.3 with statistical significance (adjusted *P* values < 0.05, “Benjamini and Hochberg” method) (Table S5). These results implied that copy number abnormality of these survival-related genes directly reflected their obvious genomic instability and their influence on gene expression during the progression of bladder cancer.

We also checked the CNVs of the genes in the two previously identified modules (corresponding to the blue and turquoise modules in Figure 4B) which had the most positive and negative correlations with the stages of bladder cancer, respectively. Interestingly, with the tumor staging, we observed that the turquoise module (in which the 93% of genes were protective) displayed significantly higher amplification CNV frequencies than the blue module (in which all genes are hazardous genes) (Figure 5B). These results seemed counteractive. A possible explanation for this phenomenon is that, the tumor cells may adopt a “self-protective mechanism”. That is, if those protective genes are completely suppressed during tumor progression, the cancer cells themselves may have problems in survival. Of course, more experimental studies will be needed to verify this hypothesis. By contrast, for the deletion CNV frequencies, the blue and turquoise module displayed an opposite trend (Figure 5C). The above results indicated that amplification and deletion of chromosomal segments can display patterns in different stage-specific gene modules during the evolution of bladder cancer. 

### 3.6. The Analysis of the DNA Methylation Related to Clinical Prognosis of BLCA Patients

As an important epigenetic regulation factor, DNA methylation has a profound and broad impact on the progression of bladder cancer. Abnormal DNA methylations in cancer cells is often associated with the dysregulation of gene expression, and thus, affect the relevant cellular physiological functions. Here, we identified several DNA methylation features of these survival-genes that may be potentially used as prognostic biomarkers of bladder cancer.

**Figure 6:**
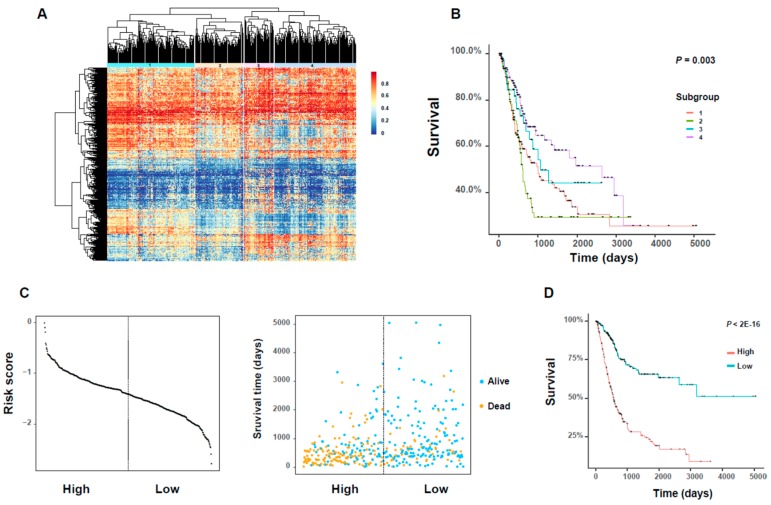
The analysis of DNA methylation profiles related to clinical prognosis of BLCA patients. (**A**) The heatmap of hierarchy clustering based on the beta values of 340 active DNA methylation probes. The rows stand for the 340 probes and the columns stand for the 404 BLCA patients. (**B**) The Kaplan–Meier plot showing the difference in overall survival rates between individual subgroups. (**C**) The distribution of the 26 gene-based risk scores (which were defined based on the 26 selected DNA methylation probes) in high- and low-risk groups (left) and the corresponding clinical characteristics of patients (right). The dotted line shows the cutoff value of the risk score. (**D**) The Kaplan–Meier survival plot of the divided high- and low-risk groups and the statistical difference between two groups of patients which was detected by log–rank test.

We first obtained 933 DNA methylation probes for the 1078 survival-related genes, whose *β*-values were the most anti-correlated with the expression of the corresponding gene (see Materials and Methods). For these 933 DNA methylation profiles, we selected the 340 ones with SD > 0.15 as “active DNA methylation probes” (see Materials and Methods; Table S6) for downstream analysis. Based on the beta values of these DNA methylation probes, we identified four subgroups with different methylation patterns by hierarchical clustering (Figure 6A; Table S6). In addition, these identified subgroups exhibited significant differences in overall survival rates of cancer patients (Figure 6B). We also performed the pairwise survival analyses between these subgroups and identified three significant pairs, i.e., subgroups 1 versus 4, subgroups 2 versus 3, subgroups 2 versus 4 (*p-*value < 0.05, log–rank test; Table S6). 

According to the clinical information of bladder cancer patients, we found that there was an obvious difference in the age distributions between the identified significant subgroup pairs (Table S6), that is, a subgroup with a higher proportion of younger patients had a survival advantage over another one with a lower proportion. More specifically, the subgroups 3 and 4 both had more than 55% of patients with ages ≤ 69 years (69 is the age median over all 404 BLCA patients), while subgroups 1 and 2 had less than 50% proportion. These results provided novel evidence for the influence of the age factor on the prognosis of bladder cancer patients.

We also applied a regularized Cox regression method, which was developed mainly based on LASSO regression, to select 26 important DNA methylation probes with non-zero coefficients from the identified 340 DNA methylation sites (see Materials and Methods; Table S6). Among these 26 significant probes, the change in DNA methylation of some members was reported to be potential indicators of some cancer types, such as *PLEKHA6* [18], *MARVELD1* [14], *MST1R* [19], *LTBP1* [20], *CLIC3* [21], *SLC13A5* [22], *SFRP5* [23], *CPXM1* [24], and *AKR1B1* [25]. After that, we introduced a risk score, which was defined as a linear combination of the *β*-values and the corresponding coefficients of these 26 DNA methylation probes derived from regularized Cox regression (see Materials and Methods). Next, we divided all BLCA patients according to the median of this new risk score, and then performed both Kaplan–Meier analysis and log–rank test on these two groups of patients. We found that the divided high- and low-risk groups displayed clearly distinct distributions of the risk score (Figure 6C). In addition, we observed significant differences between the plotted Kaplan–Meier curves, that is, the higher the risk score, the worse the prognosis, and vice versa (Figure 6D). This observation suggested that the new risk score derived from our selected DNA methylation probes can provide a good prognostic indicator for bladder cancer.

### 3.7. The Somatic Mutational Features of Survival-Related Genes 

**Figure 7:**
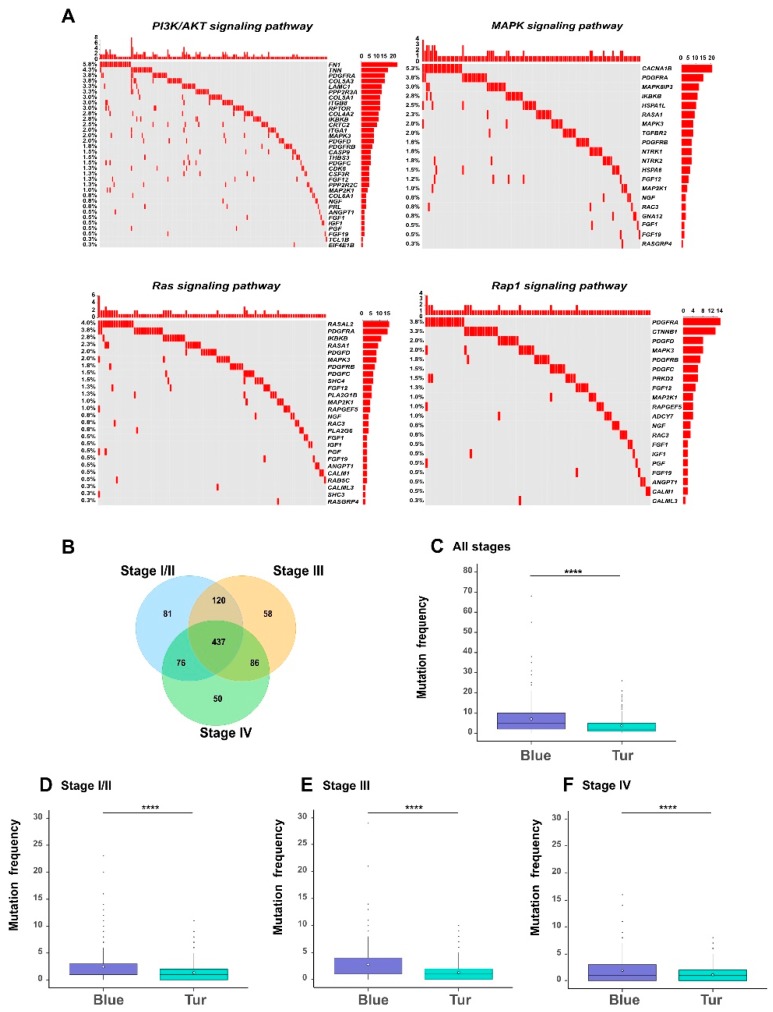
The functional signatures of somatic mutations. (**A**) The oncoprints of four examples of cellular signaling pathways that were significantly enriched with the mutated genes in BLCA samples, including PI3K/AKT pathway, MAPK pathway, Ras pathway and Rap1 pathway. Rows represent the mutated genes, which were ranked according to the frequencies of mutated genes in all samples, and columns represent the involved samples. (**B**) A Venn diagram showing the overlap of mutated genes among different stages of bladder cancer. The comparisons of the frequencies of somatic mutations between blue and turquoise modules. The white diamonds represent the mean of mutation frequency (**C**–**F**). * *p*-value < 0.05; ** *p*-value < 0.01; *** *p*-value < 0.001; **** *p*-value < 0.0001; two-sided Student’s *t*-test.

Somatic mutations often play an important role in affecting the biological functions of the related genes in cancer cells. Therefore, we profiled the genomic features of somatic mutations in the survival-related genes (see Materials and Methods). Firstly, we looked into the pathways that may be affected by the mutated genes. Through the KEGG (Kyoto Encyclopedia of Genes and Genomes) pathway enrichment analysis in DAVID database [9] on the 908 mutated genes among the 1078 genes, we noticed that a relatively large proportion (about 50%) of the enriched pathways were indeed known as tumor-related signaling pathways (Table S7). We particularly looked into four important pathways, including the PI3K/AKT pathway [26], Ras pathway [27], Rap1 pathway [28], and MAPK pathway [29], which were previously confirmed to be associated with bladder cancer. As shown in the oncoprints in Figure 7A, a considerable fraction of genes in these four pathways were mutated in bladder cancer. More specifically, 70% of the MAPK pathway, 71% of the PI3K/AKT pathway, 55% of the Rap1 pathway, and 50% of the Ras pathway had mutated genes with the frequency >1% in all samples. Our observation that these four pathways were associated with the relatively high mutation frequencies was consistent with the previous finding that mutations in the gene members of vital signaling pathways often have potential influence on the physiological function of tumor cells [30,31].

We also analyzed the distributions of mutated genes in different stages of bladder cancer (Figure 7B–F). We found that different tumor stages of BLCA patients shared a large fraction (437 genes) of mutated genes among the 1078 genes (Figure 7B). Such an observation was consistent with the previous findings that somatic mutations can have a great impact on cancer progression [32,33]. More importantly, we observed significant difference for all or stage-specific samples in the mutation frequencies between the two modules (corresponding to the blue and turquoise modules shown in Figure 4), that were the most positively and negatively correlated with tumor stages, respectively. In particular, the genes in the blue module (in which all genes were hazardous) carried more mutations than those in the turquoise module (in which 93% of genes were protective) (Table S7; Figure 7C–F). This result indicated that although somatic mutations exist in most survival-related genes, they displayed an obvious bias towards those modules of genes associated with specific properties of tumor staging. Such a phenomenon can provide useful clues into understanding the impact of somatic mutations on the evolution of bladder cancer. 

### 3.8. The Heterogeneity of MicroRNA Regulatory Networks in Different Cancer Stages

Unlike DNA methylation, CNVs, and somatic mutations, microRNAs mainly control the post-transcriptional regulation of gene expression at the RNA level. The functional roles of microRNAs in the upregulation or downregulation of gene activities in bladder cancer have been widely studied.

We particularly focused on microRNAs whose interactions with the 1078 survival-related genes were validated experimentally in previous studies. We calculated the correlation coefficients between the expression values of microRNAs and their corresponding target genes, and only selected those microRNA–gene pairs with coefficients < −0.3 as potential regulatory partners, based on which we then constructed a microRNA–gene interaction network for each stage of bladder cancer (Table S8). We found that during the progression of bladder cancer, the structure of the microRNA regulatory network tended to become sparser, including interactions involving known BLCA specific microRNAs (Figure S1). To quantitatively analyze this trend, we also calculated the assortativity degrees (see Materials and Methods) of individual microRNA regulatory networks in different stages, and observed a clearly decreasing tendency: 0.039, −0.27, and −0.27 for stage I/II, stage III, and stage IV, respectively. With respect to the biological importance, in miRNA–gene regulatory networks, the reduction of assortativity along with cancer progression means that the genes tend to lose the regulatory control of microRNAs. Here, the whole regulatory network became sparser and more discretized, which was also reflected in the number of edges in each tumor stage (Table S8). These results implied that, biologically, the dysregulation of miRNA networks becomes more serious along with tumor progression.

In summary, the microRNA regulatory networks concerning the selected survival-related genes in BLCA patients displayed a growing tendency of discretization along with the progression of bladder cancer, which can be associated with microRNA dysregulation in cancer cells. This can reflect another epitome of the disorder of intracellular regulation and control of gene expression in bladder cancer.

### 3.9. Integrative Analysis of Different Factors Related to the Staging of Bladder Cancer

**Figure 8:**
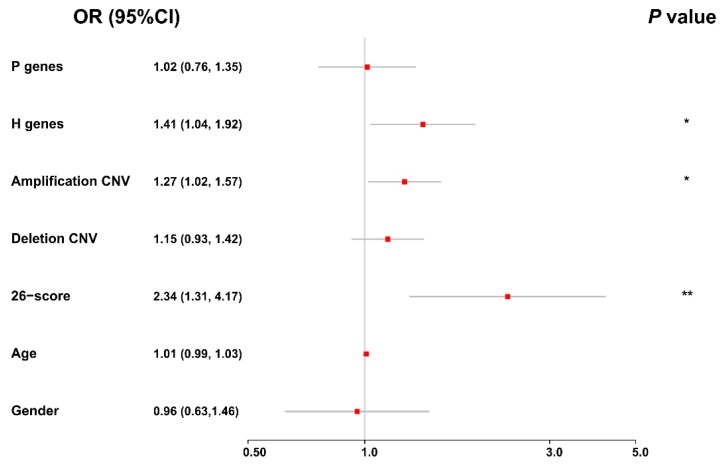
The forest plot for the ordinal logistic regression result in the integrative analysis. The red box and grey line represent the odds ratio (OR) and the corresponding 95% confidence interval, respectively, and the asterisks mark the statistically significant variables. * *p*-value < 0.05; ** *p*-value < 0.01.

To obtain a holistic understanding on the effects of different genomic and clinical factors on the evolution of bladder cancer, we further performed an integrative analysis of these factors, using an ordinal logistic regression model (see Materials and Methods). In particular, we considered the average values of the protective and hazardous genes, the frequency of copy number variations (amplification and deletion), the risk scores of DNA methylation, age, and gender in our integrative analysis. As shown in the forest plot in Figure 8, we observed that the average expression values of the hazardous genes, the frequency of amplification copy number variations, and the risk scores of DNA methylation can significantly affect the staging of bladder cancer. In addition, we found that the odds ratios (ORs) of these factors were all greater than one, indicating that they can be considered risk factors for the evolution of bladder cancer. All these integrative modeling results were consistent with the previous analyses on individual factors. Overall, in spite of the heterogeneity of genomic data derived from different platforms, the integrative analysis of multi-omics data of BLCA patients together with their clinical information, provides a high-level understanding of the joint effects of genomic and clinical factors on the evolution of bladder cancer.

### 3.10. External Validation on the Effect of the Identified Key Genes on the Progression of Bladder Cancer

To further confirm the functional effect of 1078 key genes on the progression of bladder cancer, we performed an external validation test on an independent GEO dataset (GSE13507), which contained microarray gene expression data and multiple clinical characteristics of 165 bladder cancer patients (see Material and Methods for more details). After ID mapping, we obtained 1010 genes from the GSE13507 dataset overlapping with 1078 key genes. To validate the previous analysis results obtained from TCGA data, we comprehensively analyzed the effect of these 1010 genes on the evolution of bladder cancer patients. In particular, we collected clinical information on tumor grade (high- or low-grade) and invasiveness (superficial or invasive states) of the corresponding cancer patients. After that, we performed binomial logistic regression to identify the genes that can affect the evolution of bladder cancer using gene expression values, age, and gender as the predictor variables. After correcting the *p*-values using the “Benjamini and Hochberg” method, we obtained 360 significant genes (FDR < 0.05) associated with tumor grade and 351 ones for cancer invasiveness (FDR < 0.05) among all 445 genes (Table S9, Figure S2). 

Overall, nearly half of these key genes exhibited good performance in this external validation, which greatly supported our conclusion and also reflected their potential clinical significance.

## 4. Discussion

The pathological staging of bladder cancer patients is generally associated with their overall survival. Like many other cancer types, the progression of bladder cancer is probably a multi-step and highly dynamic process, thus, it is generally difficult to evaluate the prognosis of bladder cancer only through a single gene. Therefore, a multi-level analysis of all potentially involved genes can provide a more comprehensive perspective towards understanding the evolution of bladder cancer. 

With the progression of multi-omics technology, integrative analysis of multiple genomic profiling data have received more and more attention. For example, Aine et al. used high-throughput DNA methylation arrays to screen the differential methylated regions in the whole genomic level, and then combined them with gene expression and chromosome state profiles to identify distinct DNA methylation subtypes that are associated with different epigenetic regulatory patterns in bladder carcinoma [34]. In addition, Rundstedt et al. applied target mass spectrometry to outline the alteration of metabolic signatures in bladder cancer patients, and then integrated them with the transcriptomic profiles from TCGA to detect essential metabolic pathways that are related to the progression of bladder cancer [35]. These studies mainly focused on the overall changes of one particular genomic aspect associated with the carcinogenesis of bladder cancer. On the other hand, a comprehensive analysis of molecular characterization can provide a landscape of global genomic features and elucidate the crosstalk between different genomic levels, ranging from genomic to proteomic factors. As an example, Robertson et al. [36] carried out an encyclopedic analysis on the high-throughput multi-platform sequencing data of bladder cancer and examined the genomic alteration from different angles. Through this integrative analysis strategy, they detected distinct subtypes that are closely associated with various clinical features and biological processes, such as patient survival rate, tumor progression, and critical pathways, which may offer new insights for personalized or precision therapy. Overall, these studies primarily followed a routine of “from genotype to phenotype”, that is, through systematically analyzing the multi-omics profiles and integrating genomic features that display significant differences among normal/tumor samples or among multiple-tumor-stage samples to identify specific gene targets for potential personalized diagnosis and therapy. By contrast, our study mainly used a strategy of “from top to bottom”, that is, taking the phenotypes of patients (i.e., survival status and tumor staging) as the “top features”, and directly detecting important gene candidates that are closely relevant to these features for further analyses. 

A number of previous studies have demonstrated that the tumor staging of BLCA has a crucial effect on the clinical characteristic of patients [37,38], such as overall survival time, chemotherapy response, and tumor recurrence. In addition, various molecular markers were also identified as important targets for clinical usage [39]. However, few studies have focused on the dynamic behaviors of a specific gene population in the progression of bladder cancer. In general, the cooperation among essential genes may affect the fates of cancer cells to a greater extent than the behaviors of individual genes, and thus, are more conductive to the corresponding disease phenotypes. Therefore, it is generally more appealing to comprehend the functional roles of genes within intracellular society and study their cooperative modes between each other. 

Here, the key goal of our study is to find molecular evidence that may provide useful hints on understanding the relationship between the survival of BLCA patients and tumor staging. The identified survival-related genes can serve as mediums to bridge these two important phenotypes. Technically speaking, comparing to the differential analysis methods employed in the previous multi-omics studies, our approach actually maximized a collection of survival related genes, which were useful to exploit their collaborative patterns from a more holistic perspective. In addition, unlike previous methods which mainly employed the unsupervised learning strategy to classify molecular subtypes, our framework mainly applied supervised learning techniques to identify important genes and associated crucial clinical characteristics with genomic and regulatory variation in molecular level, which may provide better accuracy and interpretability of the results.

Of course, the clinical significance of these 1078 survival-related genes needs to be further validated by more biological experiments or clinical trials, especially concerning their impacts on the progression of bladder cancer. Yet, we believe that our current study can provide useful and important insights into understanding the complex molecular mechanisms underlying the progression of bladder cancer, and thus, will be of great clinical value in the corresponding therapeutic applications.

## Supplementary Materials

The following are available online at https://www.mdpi.com/2073-4425/10/6/464/s1. Table S1: The clinical information of 404 BLCA patients obtained from TCGA, Table S2: the univariate and multivariate Cox regression results, Table S3: the functional gene modules detected by the WGCNA algorithm, Table S4: the overall connectivity and intramodular connectivity of members in the blue and turquoise modules, Table S5: the analysis results of copy number variations related to the 1078 survival-related genes, Table S6: the analysis results of DNA methylations related to the 1078 survival-related genes, Table S7: the analysis results of somatic mutations related to the 1078 survival-related genes, Table S8: the list of microRNA-gene interactions in different stages of bladder cancer, Table S9: the list of survival-related genes related to the evolution of bladder cancer based on the additional analysis from the independent GSE13507 dataset.
